# Pulmonary and Extrapulmonary Manifestations in Hospitalized Children with Mycoplasma Pneumoniae Infection

**DOI:** 10.3390/microorganisms9122553

**Published:** 2021-12-10

**Authors:** Carlotta Biagi, Alessandra Cavallo, Alessandro Rocca, Luca Pierantoni, Davide Antonazzo, Arianna Dondi, Liliana Gabrielli, Tiziana Lazzarotto, Marcello Lanari

**Affiliations:** 1 Pediatric Emergency Unit, Scientific Institute for Research and Healthcare (IRCCS), Sant’Orsola Hospital, 40138 Bologna, Italy; carlotta.biagi@aosp.bo.it (C.B.); alessandro.rocca4@unibo.it (A.R.); luca.pierantoni@aosp.bo.it (L.P.); davide.antonazzo1@gmail.com (D.A.); arianna.dondi@aosp.bo.it (A.D.); marcello.lanari@unibo.it (M.L.); 2 Specialty School of Pediatrics, Alma Mater Studiorum, University of Bologna, 40126 Bologna, Italy; 3 Microbiology Unit, Scientific Institute for Research and Healthcare (IRCCS), Sant’Orsola Hospital, 40138 Bologna, Italy; liliana.gabrielli@aosp.bo.it (L.G.); tiziana.lazzarotto@unibo.it (T.L.)

**Keywords:** Mycoplasma peumoniae, children, infants, pneumonia, respiratory tract infections, extrapulmonary manifestations

## Abstract

Mycoplasma pneumoniae (MP) is one of the main causes of both upper and lower respiratory infections in school-aged children, accounting for up to 40% of community-acquired pneumonia. Younger children are also affected, and extrapulmonary manifestations have been recently reported in the pediatric population. We carried out a retrospective analysis of MP-positive patients admitted to the Pediatric Emergency Unit of S. Orsola Malpighi University Hospital in Bologna, the largest tertiary pediatric referral center in the Emilia–Romagna region, Northern Italy, between 2012 and 2020. We identified 145 patients with MP infection (82 males and 63 females), 27% of which were younger than 2 years; the median age was 5 years (interquartile range 1–9). The clinical presentation partially differed between age groups. School-aged children were more likely to have a chest X-ray-confirmed pneumonia (*p* = 0.013), while younger children required oxygen therapy more often (*p* = 0.048). Seventy-four children (51%) showed extrapulmonary manifestations, mainly gastrointestinal (30%) and dermatological (14%). Neurological symptoms were more frequent in children older than 6 years (*p* = 0.006). The rate of other extrapulmonary manifestations did not differ significantly between age groups. This study shows that MP infection is a frequent cause of pediatric hospitalization, including of children younger than 2 years. Clinicians should be aware of the variable clinical expressions of MP, including extrapulmonary manifestations, to achieve a correct diagnosis and determine appropriate treatment.

## 1. Introduction

Mycoplasma pneumoniae (MP) is one of the main causes of respiratory tract infections, accounting for up to 40% of pediatric community-acquired pneumonia (CAP), of which 18% requires hospitalization [1]. Infections appear to be more frequent in winter and autumn, with epidemic outbreaks occurring cyclically worldwide every 3–7 years [2,3].

According to previous studies, MP involves school-aged (6–15 years old) children more frequently than younger children, who appear to be less susceptible to infection [4].

The most frequent manifestations are fever, cough (typically non-productive), and dyspnea. Other signs and symptoms that may accompany the infection are rhinorrhea, pharyngitis (typically without exudate), and otitis. Chest pain may be present, although it is less-often reported, and is attributable to the often prolonged cough [5].

According to recent literature, MP may also cause a wide variety of extrapulmonary manifestations, even without respiratory symptoms [5,6]. The incidence of extrapulmonary manifestations is 25–35%, and are mainly gastrointestinal and dermatological. Among these, in addition to the most common non-specific manifestations (vomiting, diarrhea, non-specific rash), rare cases of hepatitis, acute pancreatitis, mycoplasma-induced rash and mucositis (MIRM), and Henoch–Schonlein purpura have been reported. Neurological and cardiovascular manifestations have also been described [1,7]. In a study conducted by Gordon et al. in children hospitalized with a positive oropharyngeal swab sample for MP identified by polymerase chain reaction (PCR), 88 of 332 patients (26%) showed extrapulmonary manifestations, 39% of whom did not complain of any respiratory symptoms [8].

Moreover, recent studies showed that MP also affects pre-school children who may have a worse course of disease. This consisted in a higher hospitalization rate, the need for oxygen supplementation and intravenous rehydration requirements compared to older children [9,10,11]. On the contrary, other authors reported that the clinical severity of MP infection does not differ between age groups [8] and may be even milder in pre-school children [12].

The conflicting reports in the literature highlight the need to better investigate the epidemiological and clinical aspects of MP infection, with particular attention to the effective involvement of pre-school children, whom clinicians hardly consider the target of this infection.

The purpose of this study was to describe the epidemiology of pediatric MP infection in an Italian hospital setting over a period of 8 years and to evaluate possible age-dependent clinical characteristics, focusing in particular on extrapulmonary manifestations. Furthermore, we aimed to identify possible risk factors for the development of extrapulmonary symptoms.

## 2. Materials and Methods

### 2.1. Study Design

This was a retrospective study conducted at the Pediatric Emergency Unit (PED) of S. Orsola-Malpighi University Hospital in Bologna, the largest tertiary pediatric care center in the Emilia–Romagna region, Northern Italy. We included all children younger than 16 years of age admitted to the pediatric emergency ward between 1 January 2012 and 31 December 2020 with a positive nasopharyngeal aspirate for MP, identified by PCR. Children with two or more positive PCR-tests within three months were included only once in the study, as the infective episode was considered to be the same one. MP sampling was performed at the discretion of the medical doctor on call according to the clinical evaluation.

The primary endpoint of the study was to evaluate the clinical, laboratory, and radiological characteristics in hospitalized children with MP infection by age. Children were divided into three age groups for data analysis: less than two years old (referred to in the text as infants), 2–5 years old, and more than five years old children. Secondary endpoints were to compare demographic, laboratory, and clinical features of MP patients with and without extra-respiratory manifestations (referred to in the text as extrapulmonary manifestations), and to evaluate possible risk factors for the development of extrapulmonary symptoms.

The study was conducted in accordance with the Declaration of Helsinki, and it was approved by the ethics committee of our institution (Ethics Committee Area Vasta Emilia Centro, AVEC, approval number 531/2021/Oss/AOUBo).

### 2.2. Data Collection

Data were extracted from scanned medical charts and electronic records. They included the following information: demographic and anamnestic characteristics (age, gender, comorbidities, time between the onset of symptoms and the hospital admission, treatment before hospitalization, month of admission), clinical presentation (pulmonary and extrapulmonary signs and symptoms, body temperature and oxygen saturation), chest X-ray (CXR) and laboratory findings (blood count, C-reactive protein (CRP), creatine kinase (CK), liver function tests, anti-MP Immunoglobulin M (IgM) antibodies), bacterial or viral coinfections, medical treatment during hospitalization, and length of hospital stay.

MP testing nasopharyngeal aspirates specimens were collected, and infection was defined if the MP qualitative real-time PCR was positive. Other microbiological analyses were performed on nasopharyngeal aspirate specimens of the enrolled patients, including testing for Chlamydia pneumoniae and different respiratory viruses (respiratory syncytial virus (RSV), influenza A and B, parainfluenza virus 1-2-3, human metapneumovirus, and adenovirus). Non-vaccinated infants were also tested for Bordetella pertussis using qualitative real-time PCR on nasopharyngeal aspirate. Other bacterial pathogens were detected by respiratory cultures from either induced sputum, deep suction or bronchoalveolar lavage performed on the basis of clinical suspicion at the discretion of the pediatrician in charge. A colony count of ≥10^4^ colony forming unit (CFU)/mL was considered to be significant for bronchoalveolar lavage, while for other specimens ≥ 10^5^ CFU/mL was considered suggestive for infection. Other microbiological tests, such as blood and urine tests, were performed at the discretion of the physician in charge. 

### 2.3. Statistical Analysis

A descriptive analysis of the entire sample was made, first as a whole, then divided by age group (<2 years old, 2–5 years old and ≥6 years old). Continuous variables were synthesized as means and standard deviations (SD) or medians with interquartile range (IQR) depending on their distributive form. The qualitative variables were reported in absolute number and percentage. The distribution of the variables was evaluated by the Kolmogorov–Smirnov test. 

To compare age groups (<2 years old, 2–5 years old and ≥6 years old), ANOVA was used for the variables with normal distribution; the qualitative variables with non-normal distribution were compared using the Chi squared test or Fisher test, while the quantitative ones were compared through the Kruskal–Wallis test. In consideration of the comparison between several groups, to evaluate the most significant variable in case of significance of the ANOVA, post hoc analysis was carried out using a Bonferroni test, whereas analysis of standardized residuals was performed in case of significance of Chi-square dtest [13].

Subsequently, we compared patients with and without extrapulmonary manifestations, and patients admitted in 2014 (year of the European epidemic) with those admitted outside this period. Student t-test was used for the variables with normal distribution; the qualitative variables with non-normal distribution were compared by means of the Chi squared test or Fisher test, while the quantitative ones were compared using the Mann–Whitney test.

To evaluate the role of age in increasing the risk of extrapulmonary manifestation will be implemented multiple logistic regression models adjusted for sex, prior empiric macrolide treatment, respiratory manifestations, coinfections, and laboratory exams. The association of respiratory comorbidities and covariates with the different outcomes studied was reported as odds ratio (OR) with the relative 95% confidence interval (95% CI).

All data analyses were performed using the Statistical Package for Social Sciences (SPSS) program, version 27.0 for Windows (SPSS, Chicago, IL, USA). All hypotheses were tested using 2-tailed tests using *p* < 0.05 as the threshold of statistical significance.

## 3. Results

**Figure 1:**
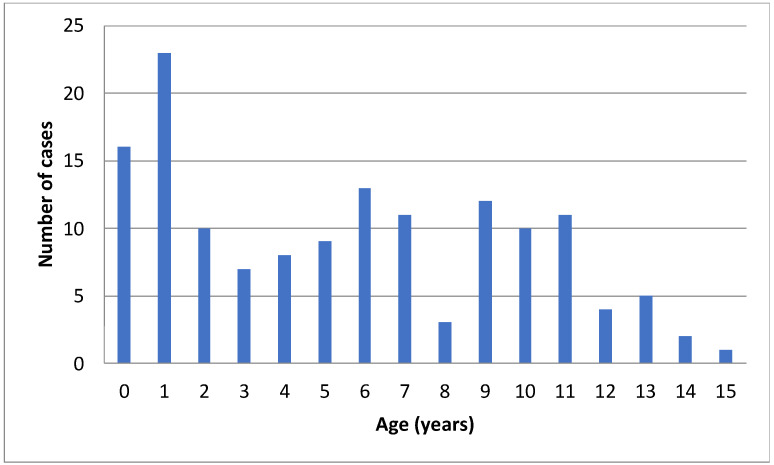
Age distribution of MP infection.

During the period between 2012 and 2020, 145 children had a positive nasopharyngeal test for MP and were enrolled in the study. Fifty-six percent were males, the median age was 5 years (interquartile range (IQR) 1–9), and 50% of the patients were younger than 6 years. The distribution of MP infection according to age, which peaked at age 0–1 year, is reported in Figure 1.

**Figure 2:**
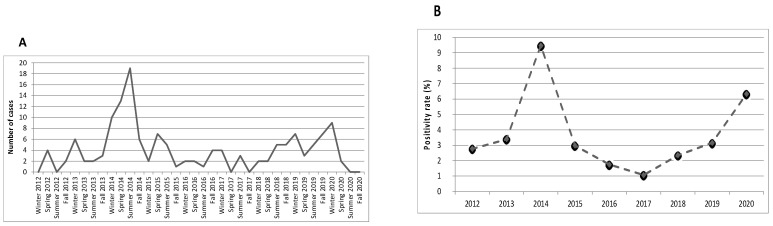
Distribution of positive MP tests during the study period. (**A**) Number of positive MP tests by months of the year. (**B**) Percent of positive MP PCR tests by year.

The distribution of positive MP tests showed a peak during 2014, with 48 hospitalized patients (33.1% of the whole cohort) and a positivity rate of 9.5% (Figure 2).

The demographic characteristics, clinical manifestations, and the laboratory and instrumental findings of the study population are summarized in Table 1.

Children were hospitalized after a median time of 7 days (IQR 4–10) from onset of clinical symptoms. Eighty-two of those (56.5%) received an empiric antibiotic treatment before hospitalization, mainly β-lactam therapy (44.1%). A total of 130/145 patients (89.7%) presented with respiratory symptoms, and 108 (74.4%) had pneumonia confirmed by CXR, associated with pleural effusion in 32 cases (22.1%). Fever and cough were the most commonly reported symptoms, respectively, in 83% and 78% of the cases. Seventy-four children (51.0%) had extrapulmonary manifestations, which were the only symptoms in 15 cases. Gastrointestinal involvement was the most common extrapulmonary manifestation (43 patients, 29.6%), represented mainly by nausea or vomiting (15.2%) followed by diarrhea or abdominal pain (6.9%). Only one patient showed transient mild transaminase elevation (aspartate transaminase 173 U/L, alanine transaminase 121 U/L) without organ damage. The second most frequent extrapulmonary manifestations were the dermatological ones (21 cases, 14.5%), particularly skin rash (11%), with a diagnosis of Henoch–Schoenlein purpura occurred in 1 case and MIRM in 3 cases. A total of 16/145 patients (11%) had neurological manifestations, mostly headache (8.3%), and one of them developed a Guillain–Barré syndrome. Concerning musculoskeletal manifestations, 6 children (4.2%) presented with arthralgia and 8 (5.5%) had myalgia associated with transient CK surges (maximum value 1257 U/L).

Laboratory analyses at admission showed a substantially normal leukocyte count with neutrophilic prevalence, and slightly high CRP (median 1.76 mg/dL; IQR 0.76–5.15). Seventy-one patients (48.9%) underwent specific serology against MP, 35 of whom (49.3%) had positive immunoglobulin M (IgM). A coinfection was found in 19 patients (13.1%). Viral coinfections were identified in 16 patients, mostly RSV (6 cases), followed by influenza and rotavirus viruses (4 and 3 patients, respectively), while bacterial coinfection was found in 3 patients (Chlamydia pneumoniae, Bordetella pertussis and Pseudomonas aeruginosa). In particular, one patient resulted positive for Chlamydia pneumonia on nasopharyngeal aspirate. This pathogen was considered a coinfection by the clinician in charge due to a documented seroconversion during the hospitalization. Moreover, two patients (non-vaccinated infants) were tested for Bordetella pertussis using qualitative real-time PCR on nasopharyngeal aspirate, and one resulted positive. Seven patients (5 with chronic pulmonary disease, 2 with immunodeficiency) were tested for other bacterial pathogens by respiratory cultures from either induced sputum, deep suction or bronchoalveolar lavage. One patient resulted coinfected by Pseudomonas aeruginosa, obtained from bronchoalveolar lavage. Three patients undergone respiratory cultures from deep suction resulted carriers of Staphylococcus aureus, Moraxella catarrhalis, and Pseudomonas aeruginosa, respectively. Macrolide was administered to 63.4% of the patients during the hospital stay. Only 18/145 patients (12.4%) needed oxygen therapy, whereas 125/145 (86.2%) received intravenous fluid therapy. The median length of hospital stay was 5 days (IQR 3.0–6.0).

### 3.1. Primary End-Point

Patient characteristics stratified by age group are reported in Table 1.

Infants were more likely to have upper respiratory manifestations with rhinitis (35.9% vs. 11.8% vs. 8.3%; *p* = 0.001) and tachypnoea (56.4% vs. 38.2% vs. 19.4%; *p* < 0.001) than older children. CXR-confirmed pneumonia was instead more common in children older than 2 years compared with infants, particularly regarding the presence of pleural effusion (2.6% vs. 32.4% vs. 27.8%; *p* = 0.013). However, during the hospitalization, infants received oxygen therapy more frequently (23.1% vs. 11.8% vs. 6.9%; *p* = 0.048).

Extrapulmonary manifestations appeared to be slightly more common in children older than 2 years, although without statistical significance (43.6% vs. 55.9% vs. 52.8%, *p* = 0.529). Analyzing the type of extra-pulmonary manifestations, children older than 5 years presented more neurological symptoms than younger children, mostly headache (0 vs. 2.9% vs. 20.8%; *p* = 0.006). The rate of the other extrapulmonary manifestations did not differ significantly between younger and older children.

Analyzing blood tests results, we found a statistically significant increase in white blood cell (WBC), lymphocytes and platelet count values in infants compared to older children, while CRP was lower in this group of patients (1.07 mg/dL vs. 2.77 mg/dL vs. 2.43 mg/dL, *p* = 0.002). Moreover, children older than 2 years had positive specific IgM serology against MP more often than younger children (12.8% vs. 29.4% vs. 27.8%; *p* = 0.043). However, considering only the patients who underwent MP serology, the percentage of positive anti-MP IgM was only slightly lower in infants compared to older children (45.5% vs. 55.6% vs. 47.6%).

Lastly, school-aged children appeared to be more frequently treated with antibiotics before (15.9% vs. 28.0% vs. 56.1%; *p* = 0.039) and during the hospitalization (35.9% vs. 58.8% vs. 80.6%; *p* < 0.001).

### 3.2. Secondary End-Points

Table 2 summarizes the demographic, laboratory, and clinical features of MP patients with and without extra-pulmonary manifestations.

Patients without extrapulmonary involvement presented more frequently rhinitis (23.9% vs. 9.5%; *p* = 0.019), cough (94.4% vs. 62.2%; *p* < 0.001) and findings on lung auscultation (83.1% vs. 66.2%, *p* = 0.020). No difference emerged regarding other respiratory manifestations, laboratory data, or radiological findings. Moreover, the rate of coinfections did not differ between patients with and without extrapulmonary manifestations. During hospitalization, patients without extrapulmonary manifestations needed intravenous fluid therapy more frequently (94.4% vs. 78.4%; *p* = 0.005), while the rate of oxygen requirement and the length of hospital stay were similar in the two study groups.

Subsequently, we compared the patients admitted in 2014 with those admitted outside this period (Table S1). In 2014, MP detection was more common in summer (37.5% of cases), whereas in other years MP infection occurred mainly in winter (33.0%) (*p* = 0.038). Patients admitted in 2014 presented more frequently findings on lung auscultation (91.7% vs. 66.0%, *p* = 0.001) and CXR-confirmed pneumonia (91.7% vs. 66%, *p* = 0.007). The rate of other pulmonary and extrapulmonary manifestations did not differ significantly between the two groups. Regarding laboratory data, no differences were founded except for statistically significant lower lymphocytes count (*p* = 0.007) and higher Hb values (*p* = 0.014) in 2014 patients. However, after comparing these values with normal ranges, they appeared to be normal. Finally, patients admitted in 2014 needed oxygen therapy less frequently (4.2% vs. 16.5%, *p* = 0.034) and had a shorter length of hospital stay (4.0 vs. 5.0, *p* = 0.001).

Univariate analysis of risk factors associated with extrapulmonary manifestations (Table 3) revealed that an empiric antibiotic treatment before hospitalization was related to increased incidence of dermatological manifestations (odds ratio (OR): 7.0; 95%; confidence interval (CI): 2.4–21; *p* < 0.001). Moreover, an elevated blood neutrophil count at admission appeared to be related with gastrointestinal manifestations (OR: 1.1; 95% CI: 1.0–1.1; *p* = 0.019).

The onset of neurological manifestations seemed to be associated with an older age of patients (OR: 1.3; 95% CI: 1.1–1.5; *p* < 0.001) and a higher value of hemoglobin count (OR: 2.4; 95% CI: 1.4–4.0; *p* = 0.001), whereas lymphocytosis was found to be a protective factor for neurological involvement (OR: 0.6; 95% CI: 0.4–1.0; *p* = 0.040), and platelets count (OR: 1.0; 95% CI: 0.9–1.0; *p* = 0.001) resulted irrelevant. Finally, leukocytosis (OR: 0.8; 95% CI: 0.7–1.0; *p* = 0.033) and neutrophilia (OR: 0.8; 95% CI: 0.6–1.0; *p* = 0.023) at admission resulted associated with a lower risk of musculoskeletal involvement.

All the risk factors associated with extrapulmonary manifestations in the univariate analysis lost statistical significance in the multivariate analysis.

## 4. Discussion

In this study, we investigated the epidemiology and clinical characteristics of children infected with MP admitted to an Italian tertiary care center between 2012 and 2020.

Previous studies have reported that, in countries with temperate climates, such as Italy, MP is detected mostly in summer and early autumn, but occurs all year [2,14,15]. According to the literature, in the present study, MP detection was slightly more common in summer (27.6% of cases), with no differences between age groups. However, the seasonal distribution of MP infections in our study was significantly influenced by the high number of cases in the summer of 2014 (Table S1). In fact, the distribution over time of MP nasopharyngeal positive tests revealed a peak in 2014, with 48/175 patients (33.1%) hospitalized in this year. This elevation in cases corresponds to an epidemic outbreak that was registered in all Europe between 2014 and 2015 [16]. 

Although this infection has typically been reported in school-age children, recent studies have shown that younger children are also affected [1,8,15,17]. In our study focused on hospitalized children, the median age of patients was 5 years (IQR 1–9), with 50% of the whole study cohort being younger than 6 years. The distribution of MP infection according to age showed a peak at 0–1 years. These results underline the importance of reconsidering the relevance of this pathogen in infants and pre-school children who may have a severe course of the disease and require hospitalization. 

Regarding clinical manifestations, the commonest symptoms in our population were fever and cough. Disease presentation differed between infants and older children; the latter being more often affected by CXR-confirmed pneumonia. In infants, respiratory tract infections can cause tachypnea and breathing difficulties even in the absence of pneumonia. In contrast, in older children, respiratory manifestations requiring hospitalization are mainly related to the presence of pneumonia. Therefore, in our study, which took place in a hospital setting, it is not surprising that infants presented more frequently with rhinitis and tachypnea, whereas CXR-confirmed pneumonia was more common in school-age children. Our data confirm what has been also described by previous studies: MP should not be considered exclusively as an agent of CAP, but also as a cause of respiratory manifestations involving the upper airways and which may determine severe clinical conditions, especially in younger children even with a negative CXR [8,15].

Regarding treatment with macrolide, it was administered at the discretion of the physician in charge on the basis of clinical pictures. School-age children appeared to be more frequently treated than infants. CXR-confirmed pneumonia was more common in older children, and this may account for the higher rate of macrolide administered to these patients. However, infants required oxygen therapy more frequently than older children, and had a higher length of hospital stay. This underlines how much the course of MP infection can be more challenging in younger children, as previously reported [9,10,11]. Our results may partly be due to the reduced use of antibiotics in infants, both before and during hospitalization, suggesting that withholding antibiotic therapy could lead youngest children to increased respiratory distress and a worse course of the disease. Nevertheless, the role of macrolide therapy in changing the clinical course of pediatric MP infection is controversial and further studies are required to investigate this aspect, as suggested by a recent Cochrane Review [18]. 

Although the respiratory system was predominantly involved, MP may also cause a wide variety of extrapulmonary manifestations. In the present study, 74 children (51.0%) had extra-pulmonary manifestations, mainly gastrointestinal and dermatological. This percentage differs from other studies which reported a frequency of extrapulmonary manifestations of 25–35% [1,7].

These data could be justified by the different criteria of patient selection: while in many previous studies only patients with radiological evidence of CAP were considered, we included all patients with positive nasopharyngeal aspirate for MP identified by PCR. This criterion may have led to the inclusion in the sample of a greater number of children with extrapulmonary manifestations but without radiological evidence of pneumonia. 

According to the literature, gastrointestinal involvement was the most common extrapulmonary manifestation, reported in 29.6% of our patients, represented mainly by vomiting, diarrhea and abdominal pain [19]. Unlike other studies, which found diarrhea and vomiting more commonly in younger children [15,20] we did not find differences in these variables. 

The second most frequent extrapulmonary manifestations were the dermatological ones, developed in 14.5% of the study population and consisting mostly of skin rash. Special attention should be given to MIRM, described in 3 of our patients. MIRM is a severe condition recently distinguished from Steven–Johnson Syndrome, characterized by exuberant mucositis with scarce or absent cutaneous involvement, which often requires corticosteroids and parenteral nutrition [21,22]. Clinicians should be aware of this condition, as a prompt diagnosis facilitates early and correct management of these patients and enables more specific counseling about prognosis. 

In line with what was reported in the literature, 11% of our patients presented neurological symptoms, especially headache, and one child presented with Guillain–Barré syndrome, a clinical condition known to be associated with MP infection [19,23,24]. The rate of neurological manifestations was the only one among the extrapulmonary symptoms that differed significantly in the three age groups (0 vs. 2.9% vs. 20.9%; *p* = 0.006), and was higher in school-aged children. This difference has been reported also by Aguilera-Alonso and colleagues, and it could be attributable to the difficulty of assessing this type of symptomatology (especially headache) in young children, as the authors have hypothesized [7].

It is worthy to note that 15/145 patients (10.3%) presented extrapulmonary manifestations without any respiratory involvement. This percentage is consistent with the one reported by Gordon et al. (11%), whose study’s selection criteria were similar to ours [8].

Regarding laboratory tests, infants had a higher number of WBC, lymphocytes, and platelets compared to older children, whereas school-age children had higher hemoglobin. Nevertheless, after comparing these values with normal ranges known for age, they appeared to be normal [25]. Similar data emerged from other studies. Gordon et al. observed higher values of WBC and platelet in pre-school children with MP infection compared with school-age ones, but all in normal ranges for age [8]. Aguilera-Alonso and colleagues reported higher WBC and lymphocytes in Spanish infants with MP-positive CAP, which however could be due to the different range of normal in this age group [7]. Finally, in an Italian study analyzing 102 hospitalized children with low respiratory tract infections due to MP, preschool-aged children had a higher number of lymphocytes, monocytes, and platelets [15].

Moreover, in our study older children had higher median values of CRP than infants, whereas no differences between age groups have been reported in the previously cited studies. However, it has to be noticed that the median value of CRP was slightly above the normal range (<0.50 mg/dL) in all age groups (1.07 mg/dL vs. 2.77 mg/dL vs. 2.43 mg/dL), with levels that are very similar to those reported in other studies [7,20].

The rate of coinfections in our study was 13.1% (viral coinfections in 16 patients, bacterial in 3 cases), with no differences between age groups. In previous studies, coinfections have been reported to be more common (29.4%), particularly in pre-school children and mostly represented by rhinovirus [12]. Our findings may be partially due to the fact that rhinovirus was not routinely tested in our patients, similarly to the study of Gordon et al. [8]. Although in some cases the co-detected pathogen may have contributed to the clinical manifestations, it is important to note that the coinfections rate did not differ significantly between patients with and without extra-pulmonary manifestations. Furthermore, coinfections were not associated with any extra-pulmonary involvement in the univariate analysis. Similar results were observed by Zhao et al., who analyzed children with CAP caused by MP in mono-detection and co-detection with other viral or bacterial agents, without finding differences in terms of clinical features and disease severity [26].

Comparing children without extrapulmonary manifestations with those with at least one extrapulmonary manifestation, the former presented more frequently respiratory symptoms such as cough (94.4% vs. 62.2%; *p* < 0.001), rhinitis (23.9% vs. 9.5%; *p* = 0.019), and abnormal breath sounds (83.1% vs. 66.2%; *p* = 0.020). Moreover, children without extra-pulmonary manifestations needed intravenous fluid therapy more often (94.4% vs. 78.4%; *p* = 0.005), probably due to respiratory distress that may lead to a diminished feeding tolerance [5].

Subsequently, a univariate analysis was performed to evaluate possible factors associated with the development of extra-pulmonary symptoms. Patient age and a higher hemoglobin count resulted as risk factors for neurological involvement, while lymphocytosis was found to be a protective factor for neurological involvement. However, all these factors lost statistical significance in the multivariate analysis. Likewise, leukocytosis and neutrophilia were found to be protective factors for musculoskeletal involvement in the univariate analysis, but lost their significance in the multivariate analysis. Conversely, an elevated neutrophil count at admission was associated with gastrointestinal involvement (OR 1.1; 95% CI 1.0–1.1; *p* = 0.019). However, this result may depend on the fact that vomiting, which is the main gastrointestinal symptom observed, is often accompanied by neutrophilia. In fact, vomiting involves activation of the stress mechanisms and mobilization of neutrophil cells to blood [27,28]. Finally, the administration of empiric antibiotic therapy before hospitalization resulted associated with the onset of dermatological manifestations (OR: 7.0; 95% CI: 2.4–21; *p* < 0.001). Thus, it is not surprising that children older than 6 years who received antibiotics more frequently before admission, presented cutaneous involvement more often than younger children, although without reaching a significant difference. The relationship between extrapulmonary manifestations and pre-hospitalization antibiotic therapy deserves to be deepened more accurately in future studies.

There are some limitations to our study. First, this is a single-center study, thus our results may not be fully representative of the epidemiology of pediatric MP infection in Italy. Moreover, the cohort of our study is based on the hospital setting and not on community setting. Thus, the findings of this study may not apply to overall pediatric population. In addition, the retrospective design of the study precluded the collection of samples for assessment of other coinfections in all patients. However, all our patients underwent microbiologic analysis on nasopharyngeal aspirate including testing for Chlamydia pneumoniae and different respiratory viruses (RSV, influenza, parainfluenza, metapneumovirus, and adenovirus) except for rhinovirus, which is not routinely performed in our hospital. Moreover, the lack of routine testing for identification of main macrolide resistance associated mutations prevented us from establishing the rate or resistance in our population. In our study, children were tested for MP infection on the basis of clinical suspicion at the discretion of the pediatrician in charge. Thus, children with MP infection without respiratory manifestations might have been underdiagnosed. Finally, a positive MP PCR test does not exclude the possibility of a past infection. However, these are common limitation of similar studies [7,8,15].

## 5. Conclusions

In this study, we found that MP infection is a common cause of pediatric hospitalization, also in children younger than 2 years. Infants present more frequently with respiratory manifestations which are less associated with CXR-confirmed pneumonia, while school-age children are more likely to have CAP. Nevertheless, younger children may have a severe course of the disease, represented in our study by a higher rate of oxygen requirement and a higher length of hospital stay. MP can also cause different extrapulmonary manifestations, mainly gastrointestinal and dermatological. In our study, only neurological involvement, mostly represented by headache, was found to be more frequent in school-age children. The rate of the other extrapulmonary manifestations did not differ significantly between younger and older children. According to this study, clinicians should be aware of the variable clinical expressions of MP, including extrapulmonary manifestations, to achieve a correct diagnosis and an appropriate treatment.

## Figures and Tables

**Table 1:** Demographic, and laboratory and clinical data of the study population.

	Total *n* = 145 (100%)	<2 Years Old *n* = 39 (26.9%)	2–5 Years Old *n* = 34 (23.4%)	≥6 Years Old *n* = 72 (49.7%)	*p*-Value
**Demographic and Anamnestic Data:**					
Sex, *n* (%), male	82 (56.6)	26 (66.7)	19 (55.9)	37 (51.4)	0.300
Background disease°, *n* (%)	34 (23.4)	10 (25.6)	7 (20.6)	17 (23.6)	0.878
Season, *n* (%)					0.079
Spring	33 (22.8)	8 (20.5)	10 (29.4)	15 (20.8)	
Summer	40 (27.6)	10 (25.6)	6 (17.6)	24 (33.3)	
Autumn	33 (22.8)	5 (12.8)	12 (35.3)	16 (22.2)	
Winter	39 (26.9)	16 (41)	6 (17.6)	17 (23.6)	
Time between symptoms onset and hospitalization, days (median, IQR)	7.0 (4.0–10.0)	5.0 (2.0–10.0)	7.0 (4.0–12.2)	7.0 (5.0–9.0)	0.150
Treatment prior to hospitalization, *n* (%)					**0.039**
No antibiotic	63 (43.4)	26 (67) *	11 (32.3)	26 (36.1)	
Empiric b-lactam	64 (44.1)	10 (25.6)	19 (55.9)	35 (48.6)	
Empiric macrolide	10 (6.9)	1 (2.6)	3 (8.8)	6 (8.3)	
Empiric b-lactam + macrolide	8 (5.5)	2 (5.1)	1 (2.9)	5 (6.9)	
**Clinical Manifestations:**					
Fever (≥38 °C), *n* (%)	121 (83.4)	31 (79.5)	27 (79.4)	63 (87.5)	0.427
Any respiratory manifestations, *n* (%)	130 (89.7)	34 (87.2)	29 (85.3)	67 (93.1)	0.396
Rhinitis, *n* (%)	24 (16.6)	14 (35.9) *	4 (11.8)	6 (8.3)	**0.001**
Pharyngitis, *n* (%)	86 (59.3)	22 (66.7)	19 (55.9)	41 (56.9)	0.248
Middle ear involvement, *n* (%)	22 (15.2)	11 (28.2)	3 (8.8)	8 (11.1)	0.060
Neck lymphadeonopathy, *n* (%)	15 (10.3)	4 (10.3)	2 (5.9)	9 (12.5)	0.430
Cough, *n* (%)	113 (77.9)	31 (79.5)	24 (70.6)	58 (80.6)	0.494
Chest pain, *n* (%)	3 (2.1)	0	0	3 (4.2)	0.212
Tachypnea, *n* (%)	49 (33.8)	22 (56.4) *	13 (38.2)	14 (19.4) *	**<0.001**
Any findings on lung auscultation, *n* (%)	108 (74.5)	28 (71.8)	26 (76.5)	54 (75)	0.892
**Extrapulmonary Manifestations:**					
Any, *n* (%)	74 (51.0)	17 (43.6)	19 (55.9)	38 (52.8)	0.529
Involvement of 1 site, *n* (%)	54 (37.2)	14 (35.9)	18 (52.9)	22 (30.6)	
Involvement of 2 sites, *n* (%)	16 (11)	3 (7.7)	1 (2.9)	12 (16.7)	
Involvement of 3 sites, *n* (%)	3 (0.1)	0	0	3 (4.2)	
Involvement of 4 sites, *n* (%)	1 (0.01)	0	0	1 (1.4)	
Cutaneous involvement:					0.138
Any, *n* (%)	21 (14.5)	2 (5.1)	5 (14.7)	14 (19.4)	
Skin, *n* (%)	16 (11.0)	2 (5.1)	4 (11.8)	10 (13.9)	
Skin + mucous membranes, *n* (%)	4 (2.8)	0	0	4 (5.5)	
Retropharyngeal abscess, *n* (%)	1 (0.7)	0	1 (2.9)	0	
Gastrointestinal involvement:					0.681
Any, *n* (%)	43 (29.6)	13 (33.3)	10 (29,4)	20 (27.8)	
Nausea or vomit, *n* (%)	22 (15.2)	7 (17.9)	5 (14.7)	10 (13.9)	
Diarrhea, *n* (%)	6 (4.1)	3 (7.7)	1 (2.9)	2 (2.8)	
Abdominal pain, *n* (%)	4 (2.7)	0	2 (5.9)	2 (2.8)	
More than 1 symptom, *n* (%)	10 (6.9)	2 (5.1)	2 (5.9)	6 (8.3)	
Serum transaminases elevation, *n* (%)	1 (0.7)	1 (2.6)	0	0	
Neurological involvement:					**0.006**
Any, *n* (%)	16 (11.0)	0	1 (2.9)	15 (20.8) *	
Headache, *n* (%)	12 (8.3)	0	1 (2.9)	11 (15.3) *	
Other symptoms (weakness, dysesthesia, dystonic movements), *n* (%)	4 (2.7)	0	0	4 (5.5)	
Cardiovascular involvement:					0.525
Any, *n* (%)	4 (2.7)	1 (2.6)	1 (2.9)	2 (2.8)	
Hypertension, *n* (%)	1 (0.7)	0	0	1 (1.4)	
Pericardial effusion, *n* (%)	2 (1.4)	1 (2.6)	0	1 (1.4)	
Myopericarditis, *n* (%)	1(0.7)	0	1 (2.9)	0	
Musculoskeletal involvement:					0.927
Any, *n* (%)	14 (9.6)	4 (10.2)	3 (8.8)	7 (9.7)	
Arthralgia, *n* (%)	5 (3.4)	2 (5.1)	1 (2.9)	2 (2.8)	
Joint swelling, *n* (%)	2 (1.4)	0	1 (2.9)	1 (1.4)	
Myalgia + serum CK elevation, *n* (%)	7 (4.8)	2 (5.1)	1 (2.9)	4 (5.6)	
Genitourinary involvement:					0.440
Macrohematuria, *n* (%)	4 (2.8)	0	1 (2.9)	3 (4.2)	
**Laboratory Tests**:					
WBC at admission (median, IQR)	10,100 (7312–14,832)	12,415 (8.932–17,020) *	10,355 (8280–14,697)	8285 (6380–13,355) *	**0.003**
Neutrophil count at admission (median, IQR)	5795 (4250–9565)	5200 (4442–9837)	6570 (4685–8590)	5760 (4200–10,062)	0.750
Lymphocyte count at admission (median, IQR)	2226 (1492–3707)	4645 (3387–5750) *	2570 (1870–3340) *	1705 (1152–2462) *	**<0.001**
Platelet count at admission (median, IQR)	325,000 (247,250–420,500)	380,500 (300,250–473,250) *	329,000 (277,250–420,750)	283,000 (230,000–379,750) *	**0.003**
Hb at admission (median, IQR)	12.4 (11.4–13.2)	11.3 (10.7–12.2)	12.0 (11.3–12.7)	13.1 (12.3–13.8) *	**<0.001**
CRP at admission (median, IQR)	1.76 (0.76–5.15)	1.07 (0.42–2.71) *	2.77 (0.24–5.10)	2.43 (0.99–6.61) *	**0.002**
Anti-MP IgM:					**0.043**
Not performed, *n* (%)	74 (51.0)	28 (71.8)	16 (47.1)	30 (41.7)	
Negative, *n* (%)	36 (24.8)	6 (15.4)	8 (23.5)	22 (30.6)	
Positive, *n* (%)	35 (24.1)	5 (12.8) *	10 (29.4)	20 (27.8)	
**CXR Findings:**					
Not performed, *n* (%)	24 (16.6)	10 (25.6)	6 (17.6)	8 (11.1)	**0.013**
Negative for lung consolidation, *n* (%)	13 (9.0)	6 (15.4)	1 (2.9)	6 (8.3)	
Lung consolidation without pleural effusion, *n* (%)	76 (52.4)	22 (56.4)	16 (47.1)	38 (52.8)	
Lung consolidation with pleural effusion, *n* (%)	32 (22.1)	1 (2.6) *	11 (32.4)	20 (27.8)	
**Hospital Course:**					
Coinfection, *n* (%)					0.070
Any	19 (13.1)	8 (21.1)	6 (17.6)	5 (6.9)	
Viral etiology	16 (11.0)	8 (21.1)	5 (14.7)	3 (4.1)	
Bacterial etiology	3 (2.1)	0	1 (2.9)	2 (2.8)	
Treatment with macrolide in hospital, *n* (%)	92 (63.4)	14 (35.9) *	20 (58.8)	58 (80.6) *	**<0.001**
Oxygen therapy, *n* (%)	18 (12.4)	9 (23.1) *	4 (11.8)	5 (6.9)	**0.048**
Intravenous fluid therapy, *n* (%)	125 (86.2)	37 (94.9)	30 (88.2)	58 (80.6)	0.105
Length of hospital stay, days (median, IQR)	5.0 (3.0–6.0)	5.0 (4.0–8.0) *	4.0 (3.0–6.0)	5.0 (3.0–6.0) *	**0.036**

Significant differences for *p*-values are indicated in bold. An asterisk (*) indicates significant differences at an adjusted alpha level of 0.008, 0.006 and 0.004 in comparison between groups for binary, ternary and quaternary variables, respectively. Background disease includes: chronic pulmonary disease, congenital heart disease, immunodeficiency or severe neurological or muscular disease. CK: creatine kinase; CRP: c-reactive protein; CXR: chest X-ray; Hb: hemoglobin; IgM: immunoglobulin M; IQR: interquartile range; MP: Mycoplasma pneumoniae; WBC: white blood cells.

**Table 2:** Demographic, laboratory, and clinical features of MP patients with and without extrapulmonary manifestations.

	Total *n* = 145 (100%)	No Extrapulmonary Manifestations *n* = 71 (49%)	Extrapulmonary Manifestations *n* = 74 (51%)	*p*-Value
**Demographic and Anamnestic data:**				
Sex, *n* (%), male	82 (56.6)	41 (57.7)	41 (55.4)	0.776
Age, *n* (%)				0.529
<2 years old	39 (26.9)	22 (31)	17 (26.9)	
2–5 years old	34 (23.4)	15 (21.1)	19 (25.7)	
≥6 years old	72 (47.9)	34 (47.9)	38 (51.4)	
Background disease *, *n* (%)	34 (23.4)	19 (26.8)	15 (20.3)	0.356
Season, *n* (%)				0.618
Spring	33 (22.8)	13 (18.3)	20 (27)	
Summer	40 (27.6)	21 (29.6)	19 (25.7)	
Autumn	33 (22.8)	16 (22.5)	17 (23)	
Winter	39 (26.9)	21 (29.6)	18 (24.3)	
Time between symptoms onset and hospitalization, days (median, IQR)	7.0 (4.0–10.0)	7.0 (4.0–11.0)	6.0 (4.0–9.0)	0.158
Treatment prior to hospitalization, *n* (%)				0.455
No antibiotic	63 (43.4)	28 (39.4)	35 (47.3)	
Empiric b-lactam	64 (44.1)	36 (50.7)	28 (37.8)	
Empiric macrolide	10 (6.9)	4 (5.6)	6 (8.1)	
Empiric b-lactam + macrolide	8 (5.5)	3 (4.2)	5 (6.8)	
**Clinical Manifestations:**				
Fever (≥38 °C), *n* (%)	121 (83.4)	61 (85.9)	60 (81.1)	0.434
Any respiratory manifestations, *n* (%)	130 (89.7)	67 (94.4)	63 (85.1)	0.068
Rhinitis, *n* (%)	24 (16.6)	17 (23.9) *	7 (9.5)	**0.019**
Pharyngitis, *n* (%)	86 (59.3)	43 (60.6)	43 (58.1)	0.842
Middle ear involvement, *n* (%)	22 (15.2)	11 (15.5)	11 (14.9)	0.740
Neck lymphadeonopathy, *n* (%)	15 (10.3)	7 (9.9)	8 (10.8)	0.584
Cough, *n* (%)	113 (77.9)	67 (94.4) *	46 (62.2)	**<0.001**
Chest pain, *n* (%)	3 (2.1)	2 (2.8)	1 (1.4)	0.535
Tachypnea, *n* (%)	49 (33.8)	26 (36.6)	23 (31.1)	0.481
Any findings on lung auscultation, *n* (%)	108 (74.5)	59 (83.1) *	49 (66.2)	**0.020**
**Laboratory Tests:**				
WBC at admission (median, IQR)	10,100 (7312–14,832)	10,050 (7782–14,877)	10,260 (6790–14,787)	0.897
Neutrophil count at admission (median, IQR)	5795 (4250–9565)	5735 (4405–8950)	5890 (4215–10,272)	0.815
Lymphocyte count at admission (median, IQR)	2226 (1492–3707)	2595 (1225–4457)	2190 (1560–3305)	0.608
Platelet count at admission (median, IQR)	325,000 (247,250–420,500)	329,000 (241,750–428,000)	313,500 (257,750–417,500)	0.783
Hb at admission (median, IQR)	12.4 (11.4–13.2)	12.4 (11.4–13.2)	12.4 (11.4–13.3)	0.503
CRP at admission (median, IQR)	1.76 (0.76–5.15)	2.26 (0.78–5.36)	1.53 (0.68–4.85)	0.350
Positive anti-MP IgM, *n* (%)	35 (24.1)	17 (23.9)	18 (24.3%)	0.792
**CXR Findings:**				
Not performed, *n* (%)	24 (16.6)	8 (11.3)	16 (21.6)	0.180
Negative for lung consolidation, *n* (%)	13 (9.0)	7 (9.9)	6 (8.1)	
Lung consolidation without pleural effusion, *n* (%)	76 (52.4)	36 (50.7)	40 (54.1)	
Lung consolidation with pleural effusion, *n* (%)	32 (22.1)	20 (28.2)	12 (16.2)	
**Hospital Course:**				
Coinfection, *n* (%):				0.540
Any	19 (13.1)	8 (11.3)	11 (14.9)	
Viral etiology	16 (11.0)	6 (8.5)	10 (13.5)	
Bacterial etiology	3 (2.1)	2 (2.8)	1 (1.4)	
Treatment with macrolide in hospital, *n* (%)	92 (63.4)	48 (67.6)	44 (59.5)	0.309
Oxygen therapy, *n* (%)	18 (12.4)	10 (14.1)	8 (10.8)	0.550
Intravenous fluid therapy, *n* (%)	125 (86.2)	67 (94.4) *	58 (78.4)	**0.005**
Length of hospital stay, days (median, IQR)	5.0 (3.0–6.0)	5.0 (3.0–6.0)	5.0 (3.0–6.0)	0.892

Significant differences for *p*-values are indicated in bold. * Background disease includes: chronic pulmonary disease, congenital heart disease, immunodeficiency or severe neurological or muscular disease. CRP: c-reactive protein; CXR: chest X-ray; Hb: hemoglobin; IgM: immunoglobulin M; IQR: interquartile range; MP: Mycoplasma pneumoniae; WBC: white blood cells.

**Table 3:** Univariate analysis of risk factors associated with extrapulmonary manifestations.

	Any Extrapulmonary Manifestations			Cutaneous Involvement			Gastrointestinal Involvement			Neurological Involvement			Cardiovascular Involvement			Musculoskeletal Involvement			Genitourinary Involvement		
	OR	IC 95%	*p*-Value	OR	IC 95%	*p*-Value	OR	IC 95%	*p*-Value	OR	IC 95%	*p*-Value	OR	IC 95%	*p*-Value	0R	IC 95%	*p*-Value	0R	IC 95%	*p*-Value
Age	1.0	0.9–1.1	0.425	1.0	0.9–1.2	0.486	1.0	0.9–1.1	0.738	1.3	1.1–1.5	**<.001**	1.0	0.8–1.3	0.924	1.0	0.9–1.1	0.985	1.2	0.9–1.5	0.176
Sex	1.1	0.6–2.1	0.776	1.5	0.6–3.9	0.374	0.5	0.2–1.1	0.088	0.8	0.3–2.2	0.612	1.3	0.2–9.6	0.789	1.8	0.6–5.6	0.282	1.3	0.2– 9.6	0.789
Prior empiric antibiotic treatment	1.0	0.7–1.5	0.981	7.0	2.4- 21.0	**0.000**	0.4	0.1–1.6	0.208	1.8	0.4–6.9	0.420	0	---	0.999	2.1	0.5–8.4	0.291	2.4	0.2–24.7	0.453
Respiratory manifestations	0.3	0.1–1.1	0.078	0.5	0.2–1.3	0.158	1.2	0.5–2.7	0.685	0.7	0.2–2.2	0.578	0	---	0.998	0.4	0.1–1.3	0.126	0.3	0.1–2.4	0.277
WBC at admission	1.0	0.9–1.0	0.806	1.0	1.0–1.1	0.626	1.1	1.0–1.1	0.062	1.0	0.9–1.1	0.887	0.9	0.7–1.1	0.443	0.8	0.7–1.0	**0.033**	1.0	0.8–1.2	0.847
Neutrophil count at admission	1.0	1.0–1.1	0.425	1.0	1.0–1.1	0.598	1.1	1.0–1.1	**0.019**	1.0	1.0–1.1	0.360	0.9	0.7–1.2	0.545	0.8	0.6–1.0	**0.023**	1.0	0.9–1.2	0.806
Lymphocyte count at admission	0.9	0.8–1.0	0.221	1.0	0.8–1.2	0.734	0.9	0.8–1.1	0.392	0.6	0.4–1.0	**0.040**	0.9	0.6–1.5	0.740	0.9	0.7–1.2	0.617	0.5	0.2–1.5	0.219
Platelet count at admission	1.0	0.9–1.0	0.805	1.0	1.0–1.1	0.279	1.0	0.9–1.0	0.611	1.0	0.9–1.0	**0.030**	1.0	0.9–1.1	0.531	1.0	0.9–1.0	0.637	1.0	0.9–1.0	0.195
Hb at admission	1.1	0.9–1.4	0.283	1.3	0.9–1.8	0.193	1.0	0.7–1.2	0.714	2.4	1.4–4.0	**0.001**	1.4	0.6–3.2	0.379	1.0	0.7–1.5	0.958	1.0	0.5–2.0	0.957
CRP at admission	1.0	0.9–1.1	0.970	1.0	1.0–1.2	0.348	1.0	0.9–1.1	0.474	1.1	1.0–1.2	0.154	0.9	0.7–1.3	0.567	0.8	0.7–1.0	0.130	1.0	0.8–1.3	0.9
Coinfections	1.1	0.5–2.5	0.780	1.1	0.3–4.2	0.873	1.3	0.6–3.0	0.531	0.4	0.1–3.3	0.399	2.3	0.2–22.9	0.490	1.1	0.3–5.4	0.899	0	---	0.998

Significant differences for *p*-values are indicated in bold. CI: confidence interval; CRP: c-reactive protein; Hb: hemoglobin; IgM: immunoglobulin M; IQR: interquartile; OR: odds ratio; WBC: white blood cells.

## Data Availability

The data presented in this study are available on request from the corresponding author. The data are not publicly available due to reasons concerning privacy.

## Supplementary Materials

The following are available online at https://www.mdpi.com/article/10.3390/microorganisms9122553/s1, Table S1: Demographic, laboratory and clinical data of MP patients admitted in 2014 and those admitted outside this period.
