# Supplementary material for: Pulmonary and Extrapulmonary Manifestations in Hospitalized Children with Mycoplasma Pneumoniae Infection
Source: Microorganisms. 2021 Dec 10;9(12):2553. doi: 10.3390/microorganisms9122553 (PMC8707942; doi:10.3390/microorganisms9122553)
Supplement: Supplementary file 1 [file microorganisms-09-02553-s001.zip › microorganisms-1466298-supplementary.pdf]

microorganisms

MDPI

Table S1. Demographic, laboratory and clinical data of MP patients admitted in 2014 and those admitted outside this period.

	**Total** *n* = 145 (100%)	**2014 patients** *n* = 48 (33.1%)	**Other years patients** *n* = 97 (66.9%)	***p*-value**
**Demographic and Anamnestic Data:**				
Sex, n (%), male	82 (56.6)	24 (50)	58 (59.8)	0.263
Background disease*, n (%)	34 (23.4)	6 (12.5)	28 (28.9)	**0.029**
Season, n (%)				**0.038**
Spring	33 (22.8)	14 (29.2)	19 (19.6)	
Summer	40 (27.6)	18 (37.5)	22 (22.7)	
Autumn	33 (22.8)	9 (18.8)	24 (24.7)	
Winter	39 (26.9)	7 (14.6)	32 (33.0)	
Time between symptoms onset and hospitalization, days (median, IQR)	7.0 (4.0-10.0)	7.0 (5.0-9.8)	6.0 (3.0-10.0)	0.348
Treatment prior to hospitalization, n (%)				0.085
No antibiotic	63 (43.4)	16 (33.3)	47 (48.5)	
Empiric b-lactam	64 (44.1)	25 (52.1)	39 (40.2)	
Empiric macrolide	10 (6.9)	2 (4.2)	8 (8.2)	
Empiric b-lactam + macrolide	8 (5.5)	5 (10.4)	3 (3.1)	
Fever (≥38°C), n (%)	121 (83.4)	40 (83.3)	81 (83.5)	0.979
Any respiratory manifestations, n (%)	130 (89.7)	46 (95.8)	84 (86.6)	0.086
Rhinitis, n (%)	24 (16.6)	4 (8.3)	20 (20.6)	0.061
Pharyngitis, n (%)	86 (59.3)	28 (58.3)	58 (97.0)	0.434
Middle Ear involvement, n (%)	22 (15.2)	3 (6.3)	19 (19.6)	0.059
Neck lymphadeonopathy, n (%)	15 (10.3)	4 (8.3)	11 (11.3)	0.659
Cough, n (%)	113 (77.9)	39 (81.3)	74 (76.3)	0.498
Chest pain, n (%)	3 (2.1)	1 (2.1)	2 (2.1)	0.993
Tachypnea, n (%)	49 (33.8)	13 (27.1)	36 (37.1)	0.230
Any findings on lung auscultation, n (%)	108 (74.5)	44 (91.7)	64 (66.0)	**0.001**
**Extrapulmonary Manifestations:**				
Any, n (%)	74 (51.0)	28 (58.3)	46 (47.4)	0.633
Involvement of 1 site, n (%)	56 (38.6)	22 (45.8)	34 (35.1)	
Involvement of 2 sites, n (%)	16 (11)	6 (12.5)	10 (10.3)	
Involvement of 3 sites, n (%)	3 (2.1)	1 (2.1)	2 (2.1)	
Involvement of 4 sites, n (%)	1 (0.7)	0	1 (1.0)	
Cutaneous involvement:				0.861
Any, n (%)	21 (14.5)	2 (5.1)	5 (14.7)	
Skin, n (%)	16 (11.0)	6 (12.5)	10 (10.3)	
Skin + mucous membranes, n (%)	4 (2.8)	1 (2.1)	3 (3.1)	
Retropharyngeal abscess, n (%)	1 (0.7)	0	1 (1.0)	

Gastrointestinal involvement:<br>Any, n (%)<br>Nausea or vomit, n (%)<br>Diarrhea, n (%)<br>Abdominal pain, n (%)<br>More than 1 symptom, n (%)<br>Serum transaminases elevation, n (%)	 43 (29.6)<br>22 (15.2)<br>6 (4.1)<br>4 (2.7)<br>10 (6.9)<br>1 (0.7)	 13 (33.3)<br>11 (22.9)<br>3 (6.3)<br>1 (2.1)<br>2 (4.2)<br>0	 10 (29,4)<br>11 (11.3)<br>3 (3.1)<br>3 (3.1)<br>8 (8.2)<br>1 (1.0)	0.376
Neurological involvement:<br>Any, n (%)<br>Headache, n (%)<br>Other symptoms (weakness, dysesthesia, dystonic movements), n (%)	 16 (11.0)<br>12 (8.3)<br>4 (2.7)	 7 (14.6)<br>7 (14.6)<br>0	 9 (9.3)<br>5 (5.2)<br>4 (4.1)	0.063
Cardiovascular involvement:<br>Any, n (%)<br>Hypertension, n (%)<br>Pericardial effusion, n (%)<br>Myopericarditis, n (%)	 4 (2.7)<br>1 (0.7)<br>2 (1.4)<br>1(0.7)	 0<br>0<br>0<br>0	 4 (4.1)<br>1 (1.0)<br>2 (2.1)<br>1 (1.0)	0.565
Musculoskeletal involvement:<br>Any, n (%)<br>Arthralgia, n (%)<br>Joint swelling, n (%)<br>Myalgia + serum CK elevation, n (%)	 14 (9.6)<br>5 (3.4)<br>2 (1.4)<br>7 (4.8)	 4 (10.2)<br>2 (4.2)<br>1 (2.1)<br>2 (4.2)	 3 (8.8)<br>3 (3.1)<br>1 (1.0)<br>5 (5.2)	0.933
Genitourinary involvement:<br>Macrohematuria, n (%)	 4 (2.8)	 1 (2.1)	 3 (3.1)	 0.727
**Laboratory Tests:**				
WBC at admission (median, IQR)	10,100 (7,312-14,832)	10,195 (6,635-13,503)	9,885 (7,625-15,335)	0.543
Neutrophil Count at admission (median, IQR)	5,795 (4,250-9,565)	6,095 (4,105-9,920)	5,735 (4,400-9,370)	0.811
Lymphocytes count at admission (median, IQR)	2,226 (1,492-3,707)	1,850 (1,215-3,068)	2,720 (1,580-4,540)	**0.007**
Platelet count at admission (median, IQR)	325,000 (247,250-420,500)	300,500 (225,000-401,000)	332,500 (258,750-430,250)	0.171
Hb at admission (median, IQR)	12.4 (11.4-13.2)	12.6 (11.7-13.8)	12.3 (11.2-12.9)	**0.014**
CRP at admission (median, IQR)	1.76 (0.76-5.15)	2.08 (0.90-4.49)	1.63 (0.71-6.21)	0.891
Anti-MP IgM:<br>Not performed, n (%)<br>Negative, n (%)	 74 (51.0)<br>36 (24.8)	 18 (37.5)<br>15 (31.3)	 56 (57.7)<br>21 (21.6)	0.072

Positive, n (%)	35 (24.1)	15 (31.3)	20 (20.6)	
**CXR Findings:**				
Not performed, n (%)	24 (16.6)	2 (4.2)	22 (22.7)	**0.007**
Negative for lung consolidation, n (%)	13 (9.0)	2 (4.2)	11 (11.3)	
Lung consolidation without pleural effusion, n (%)	76 (52.4)	29 (60.4)	47 (48.5)	
Lung consolidation with pleural effusion, n (%)	32 (22.1)	15 (31.3)	17 (17.5)	
**Hospital Course:**				
Coinfection, n (%)				0.180
Any	19 (13.1)	3 (6.3)	16 (16.5)	
Viral etiology	16 (11.0)	3 (6.3)	13 (13.5)	
Bacterial etiology	3 (2.1)	0	3 (3.1)	
Treatment with macrolide in hospital, n (%)	92 (63.4)	32 (66.7)	60 (61.9)	0.571
Oxygen therapy, n (%)	18 (12.4)	2 (4.2)	16 (16.5)	**0.034**
Intravenous fluid therapy, n (%)	125 (86.2)	45 (93.8)	80 (82.5)	0.064
Length of hospital stay, days (median, IQR)	5.0 (3.0-6.0)	4.0 (3.0-5.0)	5.0 (4.0-7.5)	**0.001**

Significant differences for p-values are indicated in bold. *Background disease includes: chronic pulmonary disease, congenital heart disease, immunodeficiency or severe neurological or muscular disease. CRP: c-reactive protein; CXR: chest x-ray; Hb: Hemoglobin; IgM: Immunoglobulin M; IQR: interquartile range; MP: Mycoplasma pneumoniae; WBC: White Blood Cells.
